# Integrated MicroRNA–mRNA Profiling Identifies Oncostatin M as a Marker of Mesenchymal-Like ER-Negative/HER2-Negative Breast Cancer

**DOI:** 10.3390/ijms18010194

**Published:** 2017-01-19

**Authors:** Giulia Bottai, Lixia Diao, Keith A. Baggerly, Laura Paladini, Balázs Győrffy, Carlotta Raschioni, Lajos Pusztai, George A. Calin, Libero Santarpia

**Affiliations:** 1 Oncology Experimental Therapeutics, Humanitas Clinical and Research Institute, 20089 Rozzano-Milan, Italy; giulia.bottai@humanitasresearch.it (G.B.); laura.paladini@humanitasresearch.it (L.P.); carlotta.raschioni@humanitas.it (C.R.); 2 Department of Bioinformatics and Computational Biology, The University of Texas MD Anderson Cancer Center, Houston, TX 77030, USA; ldiao@mdanderson.org (L.D.); kabagger@mdanderson.org (K.A.B.); 3 2nd Department of Pediatrics, Semmelweis University, H-1094 Budapest, Hungary; 4 MTA TTK Lendület Cancer Biomarker Research Group, Hungarian Academy of Sciences, H-1117 Budapest, Hungary; gyorffy.balazs@ttk.mta.hu; 5 Department of Breast Medical Oncology, Yale University, Yale Cancer Center, New Haven, CT 06520, USA; lajos.pusztai@yale.edu; 6 Department of Experimental Therapeutics, The University of Texas MD Anderson Cancer Center, Houston, TX 77030, USA; gcalin@mdanderson.org; 7 RNA Interference and Non-Coding RNA Center, The University of Texas MD Anderson Cancer Center, Houston, TX 77030, USA

**Keywords:** breast cancer, microRNAs, molecular subtypes, immune response, Oncostatin M

## Abstract

MicroRNAs (miRNAs) simultaneously modulate different oncogenic networks, establishing a dynamic system of gene expression and pathway regulation. In this study, we analyzed global miRNA and messenger RNA (mRNA) expression profiles of 17 cell lines representing different molecular breast cancer subtypes. Spearman’s rank correlation test was used to evaluate the correlation between miRNA and mRNA expression. Hierarchical clustering and pathway analysis were also performed. Publicly available gene expression profiles (*n* = 699) and tumor tissues (*n* = 80) were analyzed to assess the relevance of key miRNA-regulated pathways in human breast cancer. We identified 39 significantly deregulated miRNAs, and the integration between miRNA and mRNA data revealed the importance of immune-related pathways, particularly the Oncostatin M (OSM) signaling, associated with mesenchymal-like breast cancer cells. OSM levels correlated with genes involved in the inflammatory response, epithelial-to-mesenchymal transition (EMT), and epidermal growth factor (EGF) signaling in human estrogen receptor (ER)-negative/human epidermal growth factor receptor 2 (HER2)-negative breast cancer. Our results suggest that the deregulation of specific miRNAs may cooperatively impair immune and EMT pathways. The identification of the OSM inflammatory pathway as an important mediator of EMT in triple-negative breast cancer (TNBC) may provide a novel potential opportunity to improve therapeutic strategies.

## 1. Introduction

Breast cancer is a major cause of cancer death in women worldwide. Recent advancements in the understanding of cancer biology and oncogenic mechanisms have led to the refinement of breast cancer classification, highlighting the molecular and clinical heterogeneity of this disease. Indeed, distinct signaling pathways, genetic and epigenetic aberrations, and gene expression profiles have been associated with different breast cancer subtypes [1,2,3,4,5,6,7]. In particular, triple-negative breast cancer (TNBC), which lacks the expression of estrogen receptor (ER), progesterone receptor (PR), and human epidermal growth factor receptor 2 (HER2), encompasses a heterogeneous collection of tumors [8,9]. Specific TNBC subgroups are enriched for genes involved in the epithelial-to-mesenchymal transition (EMT) process, which has been associated with cancer progression, poor prognosis, and resistance to therapy [9]. Furthermore, cancer-related inflammation is now recognized as a hallmark of cancer, and anti-tumor immune responses are emerging as important predictors of outcome and response to therapies in TNBC [10,11]. Several molecular messengers, such as chemokines and cytokines, are able to modulate the reciprocal interaction between breast cancer cells and the immune microenvironment, thus supporting tumor development and progression [10]. Considering the complexity and heterogeneity of breast cancer, the identification of reliable subtype-specific molecules, which function to simultaneously modulate different oncogenic networks, may provide novel insights into the dynamic architecture of cancer-related pathways and an opportunity to optimize therapeutic strategies.

MicroRNAs (miRNAs) are recognized as master modulators of multiple biological and pathological processes, establishing a complex combinatorial system of gene expression and pathway regulation, as well as new therapeutic targets in different cancers [12,13]. Furthermore, recent studies highlighted the relevance of miRNAs in regulating the crosstalk between cancer and immune cells in the tumor microenvironment [14,15]. Deregulation of miRNAs expression through genetic and epigenetic alterations, impairment of the biogenesis machinery, and alterations of the tumor microenvironment (e.g., imbalance of immune cells and related cytokines), are frequently observed in human cancers, including breast cancer [12,13,14,15].

In this study, we integrated miRNA–messenger RNA (mRNA) expression profiles to identify specific pathway perturbations that characterize triple-negative/mesenchymal-like breast cancer cell lines and human breast tumors.

## 2. Results

### 2.1. Specific miRNA Expression Patterns Define Molecularly Different Human Breast Cancer Cell Lines

We analyzed the genome-wide miRNA profiles of 17 cell lines representative of different breast cancer molecular subtypes (Table S1) [16]. By selecting only miRNAs expressed in at least nine cell lines, we identified 39 miRNAs significantly deregulated between triple-negative/mesenchymal-like and non-mesenchymal-like (luminal/HER2-positive) cells (Benjamini-Hochberg-adjusted *p* ≤ 0.05). Of these, 22 and 17 miRNAs were up-regulated and down-regulated in the triple-negative/mesenchymal-like cells, respectively (Table 1).

**Figure 1:**
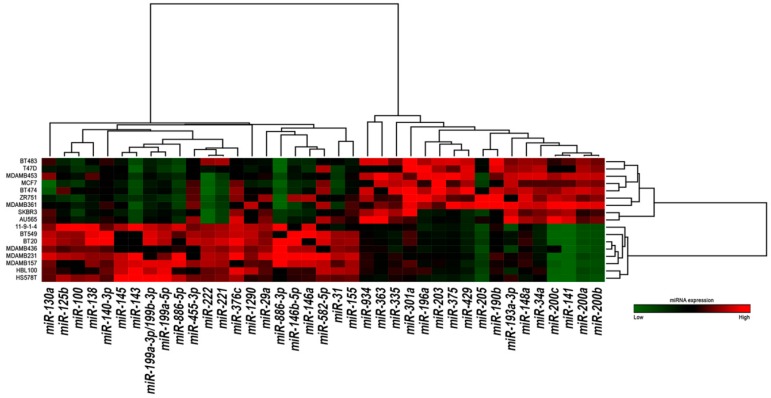
Hierarchical clustering of the 39 most differentially expressed miRNAs in 17 breast cancer cell lines. Expression values of miRNAs are represented in a matrix format, with columns indicating miRNAs and rows indicating samples. High expression values are color-coded red, and low expression values are color-coded green. Hierarchical clustering of both miRNAs (columns) and samples (rows) are shown.

Hierarchical clustering analysis revealed that the 17 breast cancer cell lines were separated into two main branches based on differences in the expression of the 39 miRNAs (Figure 1). Interestingly, the two HER2-positive cell lines (AU565 and SKBR3) formed a separate sub-cluster of the non-mesenchymal-like branch, indicating the robustness of our classification (Figure 1). These results indicate that miRNA expression profiles are able to characterize molecularly different breast cancer cell lines, and confirm that intrinsic features of breast cancer subtypes are evident also at the level of miRNAs expression.

### 2.2. Correlation between miRNA and mRNA Expression Profiles and Pathway Analysis

**Figure 2:**
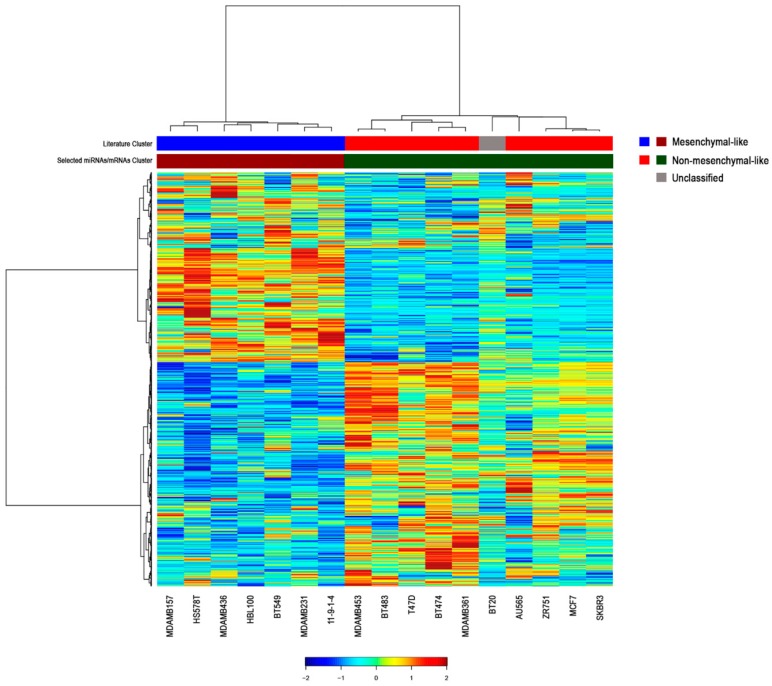
Hierarchical clustering of the most significant messenger RNAs (mRNAs) correlated with the 39 miRNAs in breast cancer cell lines. To generate the integrated profiles, we selected the mRNAs with the highest absolute correlation values for each miRNA among the 4813 genes significantly correlated with the 39 miRNAs. The color in the bars beneath the clustering illustrates the two different molecular subgroups, triple-negative/mesenchymal-like and non-mesenchymal-like (luminal/HER2-positive) subtypes. Literature cluster was assembled according to American Type Culture Collection (ATCC) and Neve et al. [16]. The unclassified BT20 cell line is depicted in grey. Hierarchical clustering of both mRNAs (rows) and samples (columns) are shown.

To investigate the role of miRNAs in the regulation of multiple transcriptomic networks, we assessed the correlation between the expression of the 39 selected miRNAs and the genome-wide mRNA profiles of the 17 breast cancer cell lines. Collectively, we found 4813 genes significantly correlated with the 39 deregulated miRNAs. Among these genes, we selected the mRNAs with the highest absolute correlation values for each miRNA using a false discovery rate (FDR) threshold of 0.05 and performed a clustering analysis of miRNA–mRNA integrated profiles (Figure 2, Table S2). The integration of expression data allowed for an accurate clustering of breast cancer cells into mesenchymal-like and non-mesenchymal-like subtypes, with the exception of BT20 cell line (Figure 2). This misclassification could be explained by the unclassified morphology of this TNBC cell line, as reported by the American Type Culture Collection (ATCC; Table S1).

Predicted and validated targets of the 39 significantly deregulated miRNAs were identified using miRBase, TargetScan, and Tarbase tools. Only a few miRNAs showed an overlap between the correlated mRNAs and the predicted targets (Table S3). Even though concordant genes may be considered as direct miRNA targets, the reduced number of overlapping genes indicates that indirect modulation mechanisms may be prevalent on direct target suppression, further strengthening the complexity and the extensive regulatory role of miRNAs on gene expression in breast cancer.

**Figure 3:**
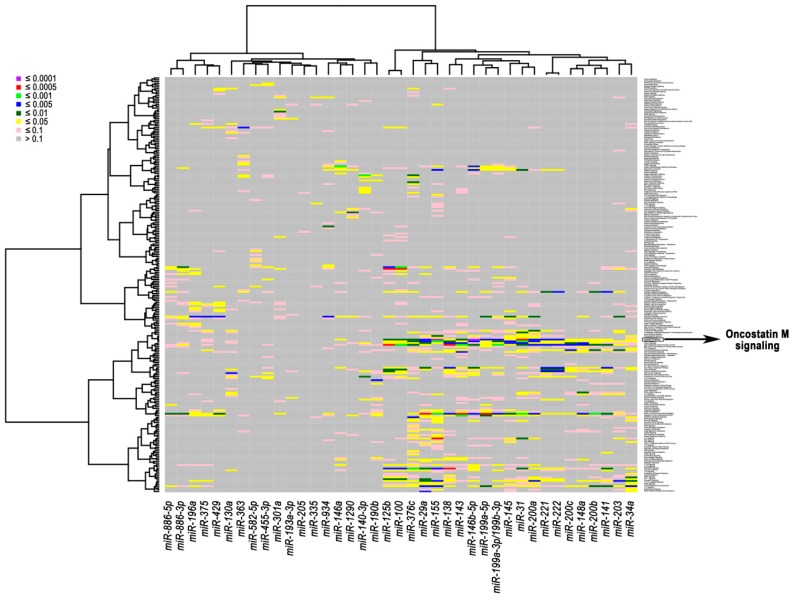
Heatmap of association between correlated miRNAs-mRNAs and signaling pathways in breast cancer cell lines. The heatmap was generated using the expression levels of the 39 selected miRNAs and correlated mRNAs. The miRNA-pathway associations were computed and displayed with eight color levels corresponding to different false discovery rate (FDR) levels.

Subsequently, we performed a pathway analysis to gain further insight into the biological functions of these miRNAs in breast cancer. The association of correlated miRNAs-mRNAs with related activated pathways is displayed in a heatmap with diverse colors corresponding to different levels of significance (Figure 3). The most significant association (FDR ≤ 1.00 × 10^−4^) was found between two miRNAs that were up-regulated in triple-negative/mesenchymal-like cells, miR-146b-5p and miR-155, and the pathway of Oncostatin M (OSM), which is a member of the multifunctional interleukin 6 (IL-6) family of cytokines (Figure 3). The OSM pathway was also consistently associated with the expression of miR-31 (FDR ≤ 5.00 × 10^−4^), and miR-29a, miR-199a-3p/199b-3p, miR-199a-5p, miR-200a, miR-221, and miR-222 (FDR level ≤ 5.00 × 10^−3^) (Table 2). Other immune-related and oncogenic pathways were found to significantly correlate with specific miRNA-mRNA networks at different FDR levels (Table 2).

### 2.3. Oncostatin M Expression is Associated with ER-Negative/HER2-Negative Breast Cancer

**Figure 4:**
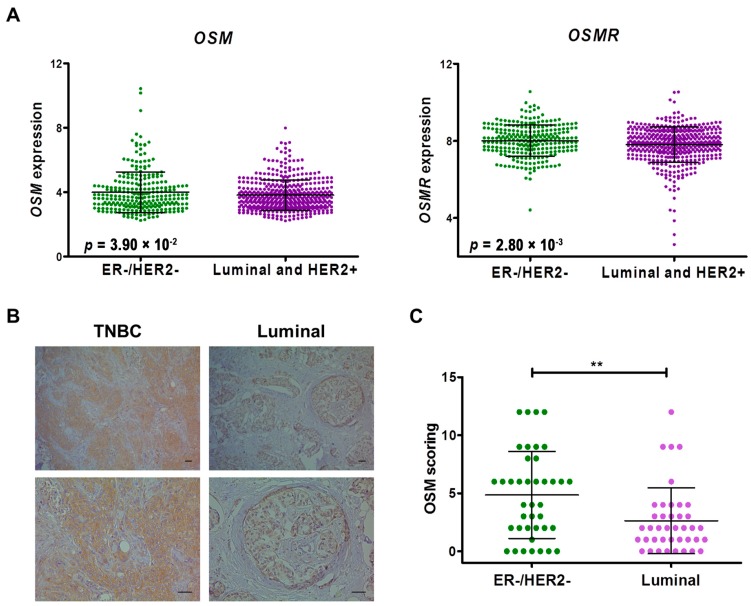
Oncostatin M (OSM) expression levels in different molecular subtypes of breast cancer. (**A**) Graphs showing *OSM* and OSM receptor (*OSMR*) expression levels in estrogen receptor (ER)-negative/human epidermal growth factor receptor 2 (HER2)-negative breast cancer and luminal/HER2-positive subtypes; (**B**) Representative immunohistochemical images of OSM expression in triple-negative and luminal breast tumors. Scale bars represent 50 µm; (**C**) Quantification of staining intensity. Data are presented as mean ± standard deviation (SD). ** *p* < 0.01.

Considering the key role of immune responses in breast cancer, we validated our findings by analyzing gene expression data from three publicly available breast cancer datasets, comprising 394 ER-positive (luminal; 56.4%), 262 ER-negative/HER2-negative (37.5%), and 43 ER-negative/HER2-positive (6.1%) breast cancers. We found that the expression of *OSM* and its receptor (*OSMR*) were significantly higher in ER-negative/HER2-negative compared with that of luminal/HER2-positive breast cancers (*OSM*, *p* = 3.90 × 10^−2^; *OSMR*, *p* = 2.80 × 10^−3^) (Figure 4A). We confirmed this finding by analyzing the expression of the OSM protein in luminal (*n* = 40) and triple-negative (*n* = 40) breast cancer samples (*p* = 6.00 × 10^−3^; Figure 4B,C).

To identify potential mechanisms mediating the effects of OSM signaling perturbation on tumor cells and the immune microenvironment, we evaluated the correlation between *OSM* expression and crucial oncogenic pathways, including inflammatory responses, EMT, epidermal growth factor (EGF), mitogen-activated protein kinase (MAPK), and phosphatidylinositol 3-kinase (PI3K) cascades. As expected, in silico analysis revealed that high levels of *OSM* expression significantly correlated with several genes associated with macrophage functions and immune microenvironment, including *ARG1*, *CCL17*, *CCL24*, *CSF2*, *CXCL11*, *GAS7*, *IL1RN*, *IL4*, *IL6*, and *NFKB1* (Table 3). Interestingly, the expression of *OSM* was also consistently associated with genes involved in EMT, EGF signaling, and downstream pathways, including *CDH1*, *EGFR*, *MAP2K7*, *MAP3K2*, *MTOR*, *PIK3R2*, *TGFB1*, and *ZEB2* (Table 3). Collectively, these results suggest that the deregulation of specific miRNA-mRNA networks could support the EMT process and contribute to the modulation of the cancer-immunity crosstalk in ER-negative/HER2-negative breast cancer.

## 3. Discussion

Numerous studies investigated the value of miRNAs as molecular classifiers, as well as diagnostic, prognostic, and predictive biomarkers in breast cancer [12,17,18,19,20]. Since miRNAs are able to modulate multiple oncogenic processes by directly and indirectly regulating gene expression networks, the integration of different -omics data represents a valuable strategy to understand the complex landscape of cancer-related pathways, and to identify novel potential therapeutic targets [21,22,23,24,25,26,27,28,29]. Accordingly, we showed that miRNA expression profiles are able to accurately characterize molecularly different breast cancer cell lines, demonstrating that a specific pattern of miRNA expression could provide important insights into breast cancer heterogeneity. Noteworthy, the integration of miRNA-mRNA expression profiles allowed for the identification of key deregulated oncogenic pathways, especially immune-related pathways, which could be involved in the development and progression of a specific breast cancer subtype. In particular, we found that the up-regulation of miR-146b and miR-155 in mesenchymal-like TNBC cell lines was consistently associated with perturbation of the OSM pathway, which has been related to reduced expression of ER and poor outcome in breast cancer [30]. Accordingly, our in silico and immunohistochemical analyses demonstrated the significant up-regulation of both the cytokine and its receptor in TNBCs compared with other breast cancer subtypes, supporting a role for the OSM signaling in this more aggressive subgroup of tumors.

Several studies demonstrated the involvement of OSM in the promotion of cell invasiveness and migration, and in the acquisition of mesenchymal-like and stem cell-like features in breast cancer [31,32,33,34]. Moreover, OSM has been suggested to promote tumor aggressiveness through the modulation of the inflammatory response, especially by inducing the recruitment of macrophages at the tumor site and the polarization toward a pro-tumor M2 phenotype [35,36,37,38,39]. Consistent with the pleiotropic role of OSM signaling in the regulation of mesenchymal-like TNBC cells-immune cells interaction, we found significant associations between OSM levels and crucial oncogenic and immune processes, including macrophage and immune functions, EMT, EGF signaling, and downstream pathways. Therefore, targeting tumor-related inflammation and the cancer-associated immune cells with pro-tumor functions may represent a novel therapeutic approach in mesenchymal-like TNBC.

## 4. Materials and Methods

### 4.1. Cell Culture, RNA Isolation, and Microarray Experiments

The breast cancer cell lines used in this study are described in Table S1. All cell lines were grown and classified according to ATCC, and as previously reported [16]. Total RNA was extracted using the standard TRIzol extraction procedures. After assessing RNA quality and yield with Agilent 2100 Bioanalyzer (Agilent Technologies, Palo Alto, CA, USA) and NanoDrop ND1000 (NanoDrop Technologies, Wilmington, DE, USA), samples were labeled with Cy5/Cy3 using a miRCURY LNA microRNA array Power Labeling kit (Exiqon, Vedbaek, Denmark), and hybridized to Exiqon miRCURY LNA miRNA array V.11.0 (Exiqon), that contains capture probes for all miRNAs registered in the miRBase version 11.0 at the Sanger Institute (available on: http://www.mirbase.org). The quality of labeling reaction and hybridization was evaluated using spike-in controls. Background corrected and log2 transformed expression data were normalized using the global locally weighted scatterplot smoothing (Lowess) regression algorithm, which we have found to produce the best within-slide normalization and minimize the intensity-dependent differences between the dyes. Gene expression profiling of breast cancer cell lines was performed using U133A Affymetrix chips containing 22,283 probe sets (Affymetrix, Santa Clara, CA, USA). Expression data were processed and normalized in the R environment using the Robust Multiarray Analysis (RMA) algorithm implemented in the Affy Bioconductor package (available on: http://www.bioconductor.org).

### 4.2. Human Breast Cancer Datasets and in Silico Analysis

To evaluate the biological crosstalk between interesting deregulated pathways in breast cancer, publicly available gene expression profiles of breast cancer patients (*n* = 699) were collected from the Gene Expression Omnibus (GEO) database (GSE25066, GSE23988, GSE20194). Gene expression data were generated with Affymetrix U133A gene chips (Affymetrix) and normalized with the Microarray Analysis Suite 5 (MAS5) algorithm, mean centered to 600 and log2 transformed using the Affy Bioconductor library [4,5]. For genes targeted by multiple microarray probes, only the probe set with the highest JetSet score was selected. ER and HER2 status were determined for each patient using the probe sets 205225_at and 216836_s_at, respectively. Normalized values >10.18 of the probe set 205225_at were considered as ER-positive, and values > 12.54 for the probe set 216836_s_at were considered as HER2-positive.

### 4.3. Tumor Samples and Immunohistochemistry

Formalin-fixed, paraffin-embedded (FFPE) tissues were retrospectively collected from patients with histologically confirmed invasive ductal luminal (*n* = 40) or triple-negative (*n* = 40) breast cancers, who underwent surgery at Humanitas Clinical and Research Institute (Rozzano-Milan, Italy). FFPE sections (3 μm) were incubated with an anti-OSM antibody (Sigma-Aldrich, Milan, Italy) for 1 h at room temperature. After washing with phosphate buffered saline (PBS), MACH 1 Universal horseradish peroxidase (HRP) Polymer (Biocare Medical, Concord, CA, USA), and diaminobenzidine (DAB; Biocare Medical) were used for chromogenic immunodetection, followed by counterstaining with hematoxylin. Negative control slides without primary antibody and positive controls were used for each immunostaining run. Images were captured using an Olympus BX53 microscope (Olympus, Tokyo, Japan). Semi-quantitative analysis of the staining intensity score was performed as previously described [40]. Briefly, the staining intensity was assessed on a scale from 0 to 3 (0, negative; 1, weak; 2, moderate; 3, strong), and staining percentage was scored from 0 to 4 (0, 0%–5% staining; 4, 75%–100% staining). A composite score was then calculated by multiplying the staining intensity by the staining percentage.

### 4.4. Statistics and Bioinformatics

Differentially expressed miRNAs, mRNAs, and proteins were identified using an unpaired *t*-test, and *p*-values were adjusted using a Benjamini-Hochberg multi-test correction as indicated in the text. Spearman’s rank correlation test was performed to evaluate the correlation between miRNAs and mRNAs. Hierarchical clustering analysis for miRNAs expression was performed using Pearson’s distance metric and average linkage using GENE-E software (available on: https://software.broadinstitute.org/GENE-E/index.html). Predicted and validated miRNA targets were identified using miRBase version 21.0 (available on: http://www.mirbase.org/), TargetScan version 6.2 (available on: http://www.targetscan.org), and Tarbase version 7 (available on: http://diana.cslab.ece.ntua.gr/tarbase). Ingenuity pathway analysis software (Qiagen, Redwood City, CA, USA) was used to define signaling pathways regulated by specific miRNAs in breast cancer cell lines. Significant miRNA-pathway correlations were identified by controlling the FDR. All tests were two-sided and the level of statistical significance was set at *p* ≤ 0.05. Statistical analyses were performed using GraphPad Prism version 5 (GraphPad Software, La Jolla, CA, USA), and R software version 3.2.2 (available on: http://www.r-project.org).

## 5. Conclusions

Collectively, our data show that specific miRNAs may have an important role as modulators of immune and mesenchymal properties in human breast cancer. The identification of deregulated miRNAs and related pathways may increase our understanding of the oncogenic functions that sustain a specific tumor phenotype and provide a potential opportunity to optimize therapeutic strategies in breast cancer.

## Figures and Tables

**Table 1:** The 39 significantly deregulated microRNAs (miRNAs) in triple-negative/mesenchymal-like breast cancer cell lines.

Up-Regulated		Down-Regulated	
miRNA	*p*-Value ^1^	miRNA	*p*-Value ^1^
miR-29a	1.52 × 10^−2^	miR-34a	5.43 × 10^−4^
miR-31	6.26 × 10^−3^	miR-141	3.08 × 10^−8^
miR-100	8.54 × 10^−3^	miR-148a	5.49 × 10^−4^
miR-125b	1.98 × 10^−2^	miR-190b	8.54 × 10^−3^
miR-130a	1.90 × 10^−2^	miR-193a-3p	6.26 × 10^−3^
miR-138	4.41 × 10^−3^	miR-196a	3.08 × 10^−2^
miR-140-3p	1.06 × 10^−2^	miR-200a	6.32 × 10^−7^
miR-143	1.06 × 10^−2^	miR-200b	3.08 × 10^−8^
miR-145	2.69 × 10^−2^	miR-200c	2.85 × 10^−7^
miR-146a	4.01 × 10^−3^	miR-203	1.81 × 10^−2^
miR-146b-5p	6.24 × 10^−3^	miR-205	2.69 × 10^−2^
miR-155	3.86 × 10^−3^	miR-301a	7.13 × 10^−3^
miR-199a-3p/199b-3p	9.11 × 10^−3^	miR-335	1.05 × 10^−2^
miR-199a-5p	2.18 × 10^−3^	miR-363	8.86 × 10^−3^
miR-221	1.88 × 10^−3^	miR-375	1.49 × 10^−2^
miR-222	2.11 × 10^−3^	miR-429	1.06 × 10^−2^
miR-376c	3.66 × 10^−5^	miR-934	3.08 × 10^−2^
miR-455-3p	1.81 × 10^−2^		
miR-582-5p	3.64 × 10^−3^		
miR-886-3p	6.24 × 10^−3^		
miR-886-5p	6.26 × 10^−3^		
miR-1290	4.41 × 10^−3^		

^1^ Benjamini-Hochberg adjusted *p*-value.

**Table 2:** The most relevant pathways related to miRNA–mRNA networks in breast cancer cell lines.

Signaling Pathway	miRNA	FDR
OSM signaling	miR-146b-5p, miR-155	≤1.00 × 10^−4^
	miR-31	≤5.00 × 10^−4^
	miR-29a, miR-199a-3p/199b-3p, miR-199a-5p, miR-200a, miR-221, miR-222	≤5.00 × 10^−3^
	miR-100, miR-125b, miR-143, miR-376c	≤1.00 × 10^−2^
	miR-34a, miR-138, miR-141, miR-145, miR-146a, miR-148a, miR-200b, miR-200c, miR-203	≤5.00 × 10^−2^
ERK/MAPK signaling	miR-138	≤5.00 × 10^−4^
	miR-376	≤1.00 × 10^−3^
	miR-100, miR-125b, miR-155	≤5.00 × 10^−3^
	miR-29a, miR-31, miR-141	≤1.00 × 10^−2^
	miR-34a, miR-146b-5p, miR-200a, miR-221, miR-222	≤5.00 × 10^−2^
JAK/STAT pathway	miR-155	≤1.00 × 10^−2^
Integrin signaling	miR-31, miR-138, miR-143, miR-145, miR-148a	≤5.00 × 10^−3^
	miR-29a, miR-200c	≤1.00 × 10^−2^
Interleukin-3 signaling	miR-155	≤5.00 × 10^−4^
	miR-31	≤1.00 × 10^−2^
	miR-29a, miR-145, miR-200a, miR-376	≤5.00 × 10^−2^
Interleukin -4 signaling	miR-31, miR-128a, miR-141, miR-148a, miR-155, miR-203	≤5.00 × 10^−2^
Interleukin -6 signaling	miR-140, miR-190b, miR-221, miR-222	≤5.00 × 10^−2^
IFN-γ signaling	miR-146b, miR-155	≤5.00 × 10^−3^
	miR-31	≤1.00 × 10^−2^
EGF signaling	miR-31, miR-145, miR-155	<5.00 × 10^−2^
T helper differentiation pathway	miR-220a	≤1.00 × 10^−2^
	miR-31, miR-130a, miR-145, miR-199a-5p, miR-203, miR-221, miR-375	≤5.00 × 10^−2^
Semaphorins signaling	miR-196a, miR-375, miR-429, miR-934	≤5.00 × 10^−3^
	miR-145, miR-199a-5p, miR-200a, miR-203	≤1.00 × 10^−2^
Leucocyte extravasation	miR-200c	≤5.00 × 10^−3^
	miR-200b, miR-203	≤1.00 × 10^−2^

Abbreviations: EGF, epidermal growth factor; ERK, extracellular signal–regulated kinases; IFN-γ, interferon-γ; JAK, janus kinase; MAPK, mitogen-activated protein kinase, OSM, oncostatin M; STAT, signal transducer and activator of transcription.

**Table 3:** Most significant correlations of *OSM* expression with cancer-related genes in ER-negative/HER2-negative breast cancers (*n* = 262).

Category	Gene	Spearman Coefficient	95% CI	*p*-Value
Macrophage function and immune response	*ALOX15*	0.442	0.34–0.54	<1.00 × 10^−4^
	*ARG1*	0.512	0.34–0.54	<1.00 × 10^−4^
	*CCL17*	0.218	0.10–0.33	4.00 × 10^−4^
	*CCL24*	0.296	0.18–0.41	<1.00 × 10^−4^
	*CD40LG*	0.335	0.22–0.44	<1.00 × 10^−4^
	*CERK*	0.196	0.07–0.31	1.50 × 10^−3^
	*CHN2*	0.282	0.16–0.39	<1.00 × 10^−4^
	*CSF2*	0.382	0.27–0.48	<1.00 × 10^−4^
	*CSF3*	0.451	0.35–0.55	<1.00 × 10^−4^
	*CSF3R*	0.460	0.36–0.55	<1.00 × 10^−4^
	*CXCL9*	−0.199	−0.32–−0.08	1.20 × 10^−3^
	*CXCL10*	−0.196	−0.31–−0.07	1.40 × 10^−3^
	*CXCL11*	−0.219	−0.33–−0.01	3.00 × 10^−4^
	*GAS7*	0.306	0.19–0.42	<1.00 × 10^−4^
	*GBP1*	−0.192	−0.31–−0.07	1.80 × 10^−3^
	*HRH1*	0.250	0.13–0.36	<1.00 × 10^−4^
	*IL1RN*	0.406	0.30–0.51	<1.00 × 10^−4^
	*IL4*	0.378	0.27–0.48	<1.00 × 10^−4^
	*IL6*	0.271	0.15–0.38	<1.00 × 10^−4^
	*IL6R*	0.232	0.11–0.5	2.00 × 10^−4^
	*IL10*	0.286	0.17–0.40	<1.00 × 10^−4^
	*IL15RA*	−0.228	−0.34–−0.11	2.00 × 10^−4^
	*IL17A*	0.280	0.16–0.39	<1.00 × 10^−4^
	*IL32*	−0.162	−0.28–−0.04	8.80 × 10^−3^
	*MERTK*	0.162	0.04–0.28	8.60 × 10^−3^
	*NFKB1*	0.385	0.27–0.49	<1.00 × 10^−4^
	*TNF*	0.376	0.26–0.48	<1.00 × 10^−4^
EMT, EGF signaling and downstream pathways	*CDH1*	−0.317	−0.42–−0.20	<1.00 × 10^−4^
	*COL1A2*	−0.338	−0.44–−0.22	<1.00 × 10^−4^
	*COL3A1*	−0.310	−0.42–−0.19	<1.00 × 10^−4^
	*DSP*	−0.227	−0.34–−0.10	2.00 × 10^−4^
	*EGFR*	0.371	0.26–0.47	<1.00 × 10^−4^
	*MAP2K6*	0.238	0.12–035	1.00 × 10^−4^
	*MAP2K7*	0.466	0.36–056	<1.00 × 10^−4^
	*MAP3K2*	0.358	0.24–0.46	<1.00 × 10^−4^
	*MAPK8*	0.33	0.22–0.44	<1.00 × 10^−4^
	*MAPK10*	0.272	0.15–0.38	<1.00 × 10^−4^
	*MTOR*	0.385	0.27–0.49	<1.00 × 10^−4^
	*PIK3R2*	0.213	0.09–0.33	5.00 × 10^−4^
	*RHOA*	0.250	0.13–0.36	<1.00 × 10^−4^
	*SRC*	0.237	0.12–0.35	1.00 × 10^−4^
	*TGFB1*	0.446	0.34–0.54	<1.00 × 10^−4^
	*ZEB2*	0.160	0.04–0.28	9.60 × 10^−3^

Abbreviations: CI, confidence interval; EMT, epithelial-to-mesenchymal transition; EGF, epidermal growth factor.

## Supplementary Materials

Supplementary materials can be found at www.mdpi.com/1422-0067/18/1/194/s1.
