# Supplementary material for: Integrated MicroRNA–mRNA Profiling Identifies Oncostatin M as a Marker of Mesenchymal-Like ER-Negative/HER2-Negative Breast Cancer
Source: Int J Mol Sci. 2017 Jan 19;18(1):194. doi: 10.3390/ijms18010194 (PMC5297825; doi:10.3390/ijms18010194)
Supplement: Supplementary file 1 [file ijms-18-00194-s001.pdf]

Supplementary Material: Integrated MicroRNA–mRNA Profiling Identifies Oncostatin M as a Marker of Mesenchymal-Like ER-Negative/HER2-Negative Breast Cancer

Giulia Bottai, Lixia Diao, Keith A. Baggerly, Laura Paladini, Balázs Győrffy, Carlotta Raschioni, Lajos Pusztai, George A. Calin and Libero Santarpia

Table S1. Characteristics of the breast cancer cell lines used in this study.

Breast Cancer Cell Line	ER Status [1]	PR Status [1]	HER2 Status [1]	Molecular Subtype [2]
AU565	NEG	NEG	POS	HER2-positive
BT20	NEG	NEG	NEG	Unclassified
BT474	POS	POS	POS	Luminal
BT483	POS	POS	NEG	Luminal
BT549	NEG	NEG	NEG	Mesenchymal-like
HBL100	NEG	NEG	NEG	Mesenchymal-like
HS578T	NEG	NEG	NEG	Mesenchymal-like
MCF7	POS	POS	NEG	Luminal
MDAMB157	NEG	NEG	NEG	Mesenchymal-like
MDAMB231	NEG	NEG	NEG	Mesenchymal-like
MDAMB361	POS	POS	POS	Luminal
MDAMB436	NEG	NEG	NEG	Mesenchymal-like
MDAMB453	NEG	NEG	POS	Luminal-androgen receptor
SKBR3	NEG	NEG	POS	HER2-positive
T47D	POS	POS	NEG	Luminal
ZR751	POS	NEG	NEG	Luminal
11-9-1-4 [3]	NEG	NEG	NEG	Mesenchymal-like

[1] Receptors status were derived from Neve, et al. [16]; [2] Breast cancer cell lines classification according to American Type Culture Collection (ATCC); [3] The 11-9-1-4 cell line is a galectin-3 transfected subclone of BT549; Abbreviations: ER, Estrogen Receptor; PR, Progesterone Receptor; HER2, Human Epidermal Growth Factor Receptor 2.

Table S2. The most significant messenger RNAs (mRNAs) correlated with the 39 deregulated microRNAs (miRNAs).

miRNA	Correlated mRNAs
miR-29a	ABCA1, ABCA2, ABR, ADAM22, AGTPBP1, AK021415, AK023582, ARHGAP22, BCAT1, BZW2, C1orf66, CAV1, CAV2, CD164, CORO1B, CYP26B1, DCUN1D1, EFNA3, EPHB2, ERI2, FEZ2, GARNL1, IL6, INPP5J, KIAA0776, LIG3, LSM5, MAGEF1, NF1, NHEJ1, NPAS2, NR2F6, PDE10A, PDE4DIP, PDGFB, PDXDC1, PHF20, PHLDA1, PLCL2, RFTN1, SEPHS2, SLC35A1, SLC6A1, SMAD3, SPTLC2, TGFBR2, TMEM186, UMPS, USP7
miR-31	ACVR1B, AF090930, ALDH3A2, ANTXR1, CCNT2, CORO1B, COX15, DBT, DHRS12, DLG3, DUS2L, DYRK3, ELF4, ERI2, FAM174B, FHL1, FOXD1, GNAS, GPR176, GRSF1, HABP4, IDE, IL6, LIG3, LINC01361, MAP2K5, MAPK13, NM_018539, PAX8, PDCD4, PHB, RANBP10, RGL2, SDC3, SHANK2, SIRT3, SOX11, SPTLC2, STAU2, STK17B, TMEM43, TNPO1, UMPS, USP7, YBX1, YTHDC1, ZNF821
miR-34a	ASCC2, B4GALT6, BAMBI, BATF3, BCL10, C1orf144, C22orf36, CASP9, CCDC40, CDC25C, CDCA8, CEP135, DVL3, EFHD2, EFS, EHBP1, GABRR1, GSTO1, ID2, ID2B, KIF1A, LSG1, MAFB, MT1F, NCOA2, OTUD4, PDZRN3, PEX26, PI4KA, PXN, PYROXD1, RAB27A, RREB1, SAMD4A, SEL1L, SLC2A11, SLC2A3, SLC7A8, SOX4, SPAG7, TBCA, TFAP2A, TFRC, TGM2, WRAP53, ZBTB11, ZMYM1
miR-100	AKAP7, ANGEL1, BBX, BOLA2, CAP1, CD59, CLIP4, CNIH3, DCBLD2, DCTN5, DNMBP, FLJ10357, FOSL2, GGA2, JUN, KIF22, KREMEN2, LEPROT, LOC101060604///SLC7A5P1///SLC7A5P2///SMG1P3, LOC730092, LPHN1, MACF1, MAP3K4, MAPRE2, NDUFAB1, NOTCH3, PARN, PARVA, PGAP2, PKP3, PMM2, PPFIA3, PPP1R15A, PTMA, RBM9, RPS15A, RRN3, RTN4, SEPT10, SERP1, SH2B1, SORBS3, TMED10, TMEM186
miR-125b	ACO1, AKAP7, BBX, BOLA2, BXDC5, C21orf33, CAP1, CD59, CLIP4, CNIH3, DCBLD2, DCTN5, DNMBP, EPPK1, FOSL2, HOXC13, JUN, KREMEN2, LEPROT, LOC101060604///SLC7A5P1///SLC7A5P2///SMG1P3, LPHN1, LPIN2, MACF1, MAP3K4, MAPRE2, NDUFAB1, NFATC2IP, NOTCH3, PARVA, PDLIM4, PGAP2, PKP3, PMM2, PPFIA3, PPP1R15A, RARG, RTN4, SEPT10, SH2B1, SORBS3, TMED10, TMEM186, TUBB3
miR-130a	AF339787, ARF5, C11orf80, C1orf116, C21orf62, CBLN1, CCPG1, CTSE, CX3CL1, EIF4G2, ELF3, FAM153A-FAM153B, HES2, ICOSLG, ITGAM, KCTD13, KLHDC2, LHFP, LUC7L2, LYN, MED8, MEF2A, MS4A2, MS4A6A, MYLIP, MYST1, NPY1R, NUP205, PLAG1, PLCH1, PPFIBP1, PRKDC, PTGER4, PTP4A1, RAG2, RPS2P45, SIRT5, SLC4A4, SLC4A7, SLC6A14, SLC6A6, SNRPN, TARP, TCF7, TM4SF1, TNPO1, TRIM3, XRCC2
miR-138	ACOX1, ARHGAP22, ATP8A1, B3GNT4, C12orf29, C7orf68, CDH11, CNIH3, DCBLD2, DDR1, EPB41L1, FLJ10357, ISLR, JUN, LSS, MAP1B, MEF2A, MPZL2, MT1E, MT1F, MT1X, NFYB, NT5E, PA2G4, PARN, PDE10A, PHC2, PIP4K2A, PLAUR, POLDIP2, PPL, RFTN1, RPL38, RTN4, SFXN3, SH2B3, SHC3, SLC5A6, SNX7, SORBS3, TMED3, TMEM186, TUBB3, UAP1, YBX1
miR-140-3p	ACTG1, AL117549, AMIGO2, AMPD2, ANK1, C15orf63, C16orf71, CTNNA1, DLST, DST, FAM129A, FAM69A, FGB, GPR27, HTRA1, ID4, IGFBP7, ITGAE, JAG1, KDELR3, MDM2, MMP14, MYNN, NOP56, OSTF1, PDCD6, PHTF1, PIGL, PLEKHO1, PMS2, REXO4, RUNX1T1, SLC5A3, SNCB, SRR, ST6GAL1, SYNE1, TARS2, TMC5, TMSB15B, TTC21B, TUBB, TWSG1, UBE4B, VPS53, WWC1
miR-141	BTN2A1, C16orf88, CD59, CLN3, CTSB, CUTA, DEPDC1, DPYD, ECHDC1, ELK3, EPHA1, FADS1, FAM21C, FGFR4, GBE1, GTF3C2, HMGA2, ID2, IRX4, LIG3, MICA-MICB, NAV3, NR4A1, PALM2-AKAP2, PAM, PEA15, PEX26, PHLDA1, PI4K2A, PICALM, PIK3R3, PLA2G3, PMM2, RAB27A, RAB3A, RABEP2, RTN4, SAMD4A, SEPT10, SIRT3, SOX4, TNFRSF10B, TPST1, TRIM3, USP7
miR-143	ACE2, ANK3, ARMC9, ASB8, ATP6V1G1, C10orf116, C19orf10, CAV1, CAV2, CCDC88A, CDH11, CLMN, COL3A1, COL6A2, DDR1, DGKZ, DSE, ELF3, FAM127A, FHL1, FLJ10357, KDELR3, LSS, MAP1B, MAPKAPK5, MGC87042, MLL, MRC2, MRPS35, MRS2, NRP2, NUAK1, ORAI2, PPFIBP1, PPL, PTK6, RALGPS1, RBM47, SNX7, SORL1, ST14, TIMP1, TMEM45A, TOMM70A, TPD52, TPM2, TRAF4, TUBB3, ZFX

Table S2. *Cont.*

miRNA	Correlated mRNAs
miR-145	ARHGAP8, ATP6V0E1, C14orf139, C14orf147, CCS, CORO1B, E4F1, ERI2, ESRP1, ETFB, FAM117A, FAM86A, FASN, FBXW2, FEM1B, GARNL1, GGA1, HIRIP3, HN1L, INPP5J, KIAA0776, L2HGDH, LIG3, MAGEF1, MMP17, NBEAL2, NFATC3, NUAK1, NUBP2, ORAI2, PDGFB, PDS5A, PDSS2, PER2, PHB, RPS6KB2, RUSC2, SEC16A, SFI1, SLC35A1, SLC48A1, SLTM, SNAI2, SON, STEAP3, SYN2, TMEM106C, ZNF652
miR-146a	AA521034, AFAP1, ARHGAP22, AVPR1A, BCL9, BCS1L, BICD2, C19orf29, CASQ1, CCL5, DDX58, DDX60, DHCR7, DPAGT1, DPYSL3, EPHB2, FGFR1, FTL, GNRH1, HLA-G, HMGCR, IFIT1, IL1B, IRF1, IRF6, KCTD12, LBH, MUC1, N4BP2L2, NACA2, NF1, NUFIP1, PKN1, PLCL2, PMAIP1, RHOT2, RIOK3, RPL7P25, SCG3, SEPT4, SMA4, SPIN1, SRP54, TCEB3, XKR8, YWHAQ
miR-146b-5p	ALDH3B2, ARHGAP8, ARPC5, ATP2C2, C1orf123, C4orf29, CAPG, CDS1, CEACAM6, CUTA, DDX60, E4F1, EMP3, FASN, GALNT3, HDDC2, ID2B, IFIT1, IFIT3, IL6, KIAA0556, LASS6, MAP7, NFATC3, NUP98, OPTN, ORAI2, PDLIM7, PDSS2, PHLDA1, PI4K2A, PROS1, PRSS8, PTTG1, RHOT2, SGPL1, SLC35A1, SLC48A1, SMARCA1, SNAI2, SPDEF, TAF7L, TMCC1, TPD52, TRPC1, VAT1
miR-148a	ABCF3, AC068039.4, ACE2, AKAP12, ARMCX1, B3GNT4, CFLAR, CSRP2, CUL4B, DKFZP586I1420, DST, FADS1, FAM127A, FERMT2, GAS2, LGALS1, LOC29034, LYZ, MARK2, MICA-MICB, NAALADL1, NLE1, OSMR, PLA2G3, PPFIBP1, PPIL2, PSIP1, RAB27A, RTN4, SERHL2, SETD6, SIRT3, SLC7A8, SRR, STX2, TMEM90B, TRIM3, TRPC1, TTLL12, UCP2, ZMYM6
miR-155	ABCA2, AMFR, BAT2L, CBR4, CBX5, CD48, CDC14B, CTTN, DCXR, DHRS12, EFEMP2, ENTPD1-AS1, ERI2, ETS2, FHL1, FKBP3, FN3KRP, GPR176, GRAMD4, HABP4, HIF1AN, HLTF, HOXC10, IDE, LDB1, LIG3, LIMS1, LRBA, LUC7L3, NCAM2, NPC1, NUBPL, PDCD4, PIAS1, PSMD12, RAB11A, RP11-473I1.9, SAPS3, SEC31A, SHANK2, SIRT3, SLC16A5, ST3GAL2, TBCD, TGOLN2, TIMP3, TNFRSF4, TSPAN13
miR-190b	ACE2, ADARB2, AK025278, ATP6V1G1, C5orf66, CA12, CFTR, CLIC1, CUL4B, CX3CR1, CYP2B6, CYP2C9, DNAH7, ENSG00000214179, GAS2, GML, Hs.677410, HTR4, IKZF1, IL11, IL1F5, JMJD7-PLA2G4B///PLA2G4B, KDM4C, LCN2, LOC29034, LYZ, MAGIX, MARCH1, NODAL, ODAM, ORAI2, PCDHB17, PCOLCE, PRINS, RHO, SLC7A8, SOD3, SOX10, THPO, TNNI3K, TRMT61A, ZNF132, ZNF816A, ZSCAN18
miR-193a-3p	AL161956, ASPH, AZGP1P1, BSDC1, CCR1, CD36, CIB1, CIC, CLINT1, CLPTM1, CLSTN2, CNPY3, CYP2C9, DNAH7, ECD, FAM149B1, FAM169A, FBXO9, FCER1A, FCF1, FLRT3, FZD4, GNPDA1, GPR126, GRLF1, HPSE, IGHA1///IGHD///IGHG1///IGHG2///IGHG3///IGHM///IGHV4-31, IPO7, IVNS1ABP, LOC100507577///LONP2///SIAH1, MCTS1, MRPL23, MYH7, NDUFA1, NM_018033, NPR3, OPRM1, RANBP17, RNF113A, RP11-345P4.4, RP1-272E8.1, RPS6, SHFM1, SMAD4, SNUPN, SPAG6, STAM2, ZFPL1, ZNF350
miR-196a	ACADM, ADAMTSL3, AF052103, AK025116, AKAP8L, BCL10, C6orf47, CCDC40, CCNE2, CNTNAP2, CTSL1, DNAH7, ECD, EXPH5, F7, FAM13C, FAM149B1, FAM86C, GATAD1, HOXC10, IGHA1///IGHD///IGHG1///IGHG2///IGHG3///IGHM///IGHV4-31, KLHDC3, LYZ, MXD4, NDUFA1, OPRM1, OR6A2, OTUD3, OTUD4, PAK2, PAX3, PCGF3, PDE4D, POPDC3, RAB1B, RGS17, RP11-345P4.4, RP11-649A18.7, RPS4X, SNX29, STK10, TCP11L1, TFAP2A, TFPI2, TMEM39B, TTLL1, ZNF24
miR-199a-3p/199b-3p	ARHGAP8, ARPC5, C6orf145, CD97, CEBPA, CHST8, CNP, CORO1B, CRTAP, CTCF, CUTA, E4F1, EHBP1, ESRP1, FAM174B, FAM21A, FEM1B, FUT9, GRAMD4, HSPA13, IL32, IL6ST, KIAA0556, LASS6, LMNA, MIPEP, NFATC3, NPDC1, PANK3, PATZ1, PRKAA1, PTRF, RABEP2, RABGAP1L, RANBP10, SAP18, SETD6, SHANK2, SLC35A1, SNAI2, SPDEF, STK17B, TIMP2, TMEM43, TRPC1, UPP1, WIPF1

Table S2. *Cont.*

miRNA	Correlated mRNAs
miR-199a-5p	ARPC5, ATP7B, C2orf34, CD97, CLCN2, CTAGE5, E4F1, EFNA3, EHBP1, EPN3, FADS3, FASN, FEZ2, FUT9, FXYD3, FYN, GCHFR, HN1L, IL6, IRF1, KDM2A, KIAA0556, LASS6, MIPEP, MPHOSPH6, NBEAL2, NFATC3, NOL3, NPDC1, PDGFB, PIK3CD, POLE, PPP2R4, RABEP2, RHOT2, RNF208, SERPINE1, SETD6, SHANK2, SLC35A1, SLC48A1, SNAI2, STAT5B, TGFB1, TRPC1, TSPAN13, ZNF692
miR-200a	ABCF3, ACVR1B, C11orf80, CORO1B, DCUN1D1, DLG3, DYRK3, EHBP1, ELF4, ELL3, FAM174B, FBXO9, FBXW2, FECH, GFPT2, IL13RA1, LIG3, LRRC1, MARK2, NAV3, NUAK1, NUDT4, NUDT4, OSMR, PFN1, PMP22, PPFIBP1, PPIL2, PSIP1, RGL2, RREB1, SDC3, SLC7A8, SOX11, SPDEF, STK10, STK17B, TFPI2, TMEM39B, TMEM43, TMEM45A, TNPO1, WNT5B, ZFX, ZMYM6, ZNF24
miR-200b	ACVR1B, ARSE, C11orf80, CDKN2C, CUL4B, CUTA, DPYSL2, ELF4, ELK3, ENSG00000212670, ERI2, FAM174B, FERMT2, FGFR4, FN1, GBE1, HEG1, HOOK2, ID4, ITGA5, LSR, MARK2, NAV3, PAK4, PARD6A, PLA2G3, PMS2, PPCS, QSOX1, RANBP10, RMND5A, RREB1, SEMA4A, SEPHS2, SIRT3, SMC5, SOAT1, SOX4, STK17B, TBC1D1, TMEM43, TMEM45A, TNPO1, TSKU, UAP1, USP7, WWTR1, ZNF768
miR-200c	ABHD2, ACE2, ARHGEF16, B3GNT4, BTD, BTN2A1, CUL4B, DEPDC1, DPYD, DST, FADS1, FAM114A1, FAM129A, FZD7, GPM6B, ICOSLG, KANK2, KHK, LOC29034, MCF2L2, MICA///MICB, NAALADL1, NLE1, NR4A1, PAM, PKP3, PLA2G3, PPIL2, PRPF8, PTPRG, RAB27A, RPA1, RTN4, S100A8, SERHL2, SLC7A8, TPD52, TPST1, TRAF4, TRPC1, TTLL12, UAP1, ZG16, ZMYM6
miR-203	ABCG1, ACSL4, ARHGAP28, BAT2, BCL10, BCOR, C5orf30, CDC14B, CGREF1, EHMT2, EIF4ENIF1, ELF4, ENSG00000212670, FAM13C, FBXO9, FECH, FN3KRP, GBP1, HHEX, HOXC10, ID2B, IL13RA1, LPHN1, LPIN1, MXD4, NAV3, NDUFC2, NFIB, NXPH3, PAK2, PDE4D, PLCE1, PRMT2, PTEN, RAB3GAP1, RHOT1, SENP5, SEPW1, SLC9A6, SP140L, SS18, ST3GAL2, TFAP2A, TFPI2, VPS4B, ZFHX4
miR-205	ACTR3, C17orf71, C6orf27, CEP250, COG8, COIL, CTC-338M12.4, DOLK, DR1, EIF3I, ESR1, FAM63B, FNDC3A, GABARAPL2, H2AFY2, HEATR6, HLA-DRB4, IGF1R, KIAA0174, LAPTM4B, LIMCH1, LRRC14, MAN1A1, MDK, MED13L, MKS1, MLYCD, MTHFSD, NEUROD1, NFATC1, OGN, PARP6, PDK3, PSKH1, PTRH2, SBF1, SF3B3, SMARCD1, TMEM165, WDR59, YWHAZ, ZNF512B, ZNF91
miR-221	AK021744, ARAP2, BAZ1A, C5orf13, C7orf68, CD59, CDKN2C, CHST3, CNIH3, CTSB, CYP26B1, DPYSL2, EFNA1, ELF3, ERBB2, EXD2, FAM21C, HLA-A, IQGAP1, LGALS1, LSS, LYN, LYPD3, METTL2B, MPZL2, PHLDA1, PICALM, PKP3, PLA2G3, PMM2, PNMA2, PVRL3, PXN, RGS20, RTN4, S100A8, SFXN3, SPTBN1, TMED3, TUBB3, TUFM, UBD, ZEB1, ZNF768
miR-222	AK021744, ARAP2, BAZ1A, C5orf13, C7orf68, CD59, CDKN2C, CHST3, CNIH3, CTSB, CYP26B1, DPYD, DPYSL2, EFNA1, ELF3, ELK3, ERBB2, EXD2, FAM21C, HLA-A, IQGAP1, LGALS1, LYN, LYPD3, METTL2B, MPZL2, PHLDA1, PICALM, PLA2G3, PMM2, PNMA2, PVRL3, PXN, RGS20, RTN4, SFXN3, SPTBN1, TLE2, TMED3, TUBB3, TUFM, UBD, ZEB1, ZNF768
miR-301a	ACAD10, AK024213, ANKLE2, ANKRD10, BCOR, CACNA2D2, CDC6, CHAT, COPG, DEAF1, DNAJC4, DVL3, ENSG00000244692, EPB41, F7, GABARAPL3, GCN1L1, GPR98, HAL, HPSE, HRH4, Hs.671977, HTT, INADL, KPNA1, LMTK2, LSM14A, MXD4, NPIPA1, PDE4D, PGR, POM121, RABGAP1, RASA1, RHOT1, RPL29, RSL1D1, SETD2, SLC1A4, SYNGR1, THBS3, TRIM2, TTLL1, WDR5B, WNK1, ZFP36L1
miR-335	ABCC3, ANAPC13, ARL17, ATG2B, ATP6V0E1P2, B4GALT6, BAT2, BTF3, C19orf2, C19orf56, C1orf21, C6orf62, CASP2, CDK5R1, CEP76, CFLAR, CSNK1D, CYP21A2, EPB41L3, FEN1, GAD2, Hs.660870, IFRD1, IL13RA1, KCNJ5, LRP3, LRRC16A, MAK, MTMR3, NDUFA2, NDUFS5, ORC4L, PACRG, PEX10, PKNOX1, PLD1, PPP2R1B, PRPF4, RAB3GAP1, RIF1, RXRG, SMAD2, SMAD4, SMAD6, TM2D1, TNFSF11, X79200, ZNF117, ZNF146
miR-363	AF052172, ANKHD1, ARIH1, ARMCX2, BCL7B, C11orf58, C1orf89, CD28, CFLAR, CLIC5, CLK3, CTDSPL, DMPK, DNAH2, DNAH7, DOCK2, EDC3, ELAC1, ERN1, FSTL4, GAS2L1, GRIP2, GSTM1, HMG20A, HMMR, Hs.589088, ING4, INTS7, LOC101929726, MIS12, MLF1, MR1, MS4A2, MTHFS, NINL, OR2B2, PPFIA1, PPFIBP2, PTGER3, PTGIR, RGS17, RHAG, RP4-621B10.8, SEC11A, SGSH, SLC34A1, SOBP, TMEM63A, UHRF1BP1L

Table S2. *Cont.*

miRNA	Correlated mRNAs
miR-375	ABCC4, ABCG1, C5orf30, C6orf47, DKFZP586I1420, EFS, EIF4ENIF1, ELF4, FBXO9, GFPT2, GGA1, HADH, HHEX, HPSE, IL13RA1, LASS6, LIMK2, MAFB, MAPK13, MYNN, NFIB, NUDT4, OPRM1, PARD6A, PPCS, PPFIA1, PPFIBP2, PSIP1, RREB1, S100A10, SEC61B, SEPW1, SLC7A8, SOX11, SP140L, SPAG7, SPDEF, SS18, TBC1D1, TCP11L1, TFPI2, TRIM2, TSPAN3, U43604, WRAP53, ZNF24
miR-376c	ADAM12, ALDH3A2, ANKH, ARNTL2, ATXN2L, BCOR, C10orf18, C1orf35, CCR4, CDH11, CNOT6, COG2, CSF1, CUL3, CYP2C9, DUSP3, ERCC1, FGF5, FN3KRP, FOSL2, HARS2, Hs.554262, ICT1, IFNA4, KIAA0485, MAP4, MAP4K3, MAPKAPK5-AS1, NUCB1, PAX8, PDE1C, PRPSAP1, RAB4A, RBMS1, RGS4, RUNX1, SH3BP2, SIRT7, SKI, SLC23A2, SNAPC5, TBCD, TLL1, ZBTB20, ZFAND1, ZFHX4
miR-429	AAGAB, ADAMDEC1, ALPL, ASCC1, C17orf39, C5orf30, C6orf47, CDC6, DHRS12, ERO1LB, ERP44, FBXW2, H2AFY, HEY1, HPSE, HTT, IGHA1///IGHD///IGHG1///IGHG2///IGHG3///IGHM///IGHV4-31, KIAA0562, KNTC1, L1CAM, LMO4, LOC100131689, MAPK13, MOGS, MXD4, NM_018539, OPRM1, PARD6A, PCGF3, PIGN, PRKY, RAB31, RANBP10, RASL11B, RPL23, RPS4X, SEC61B, SHANK2, SOX11, STK10, TBC1D1, TCP11L1, TFPI2, TRIM2, TSSC4, U43604
miR-455-3p	ANK2, APBA1, ASCL3, ATF7IP, BRWD2, CA5B, CARD9, CCDC30, CHST10, COL3A1, CYP2A6, DCLK2, ECT2, EEF1E1, FAM48A, GTPBP1, GZMK, HBB, HNF4G, Hs.670136, Hs.677353, HS3ST2, LALBA, LOC90925, M84605, METTL2A///METTL2B, MRS2, MYCBP, NCAM1, NDUFA5, NFAT5, PARL, PFDN6, PIGH, PKNOX2, PLA1A, POLR1C, PRKG1, PTPRB, PTPRN2, RAB7A, RC3H1, RHD, SCAPER, SIGLEC5, SLCO2B1, SSPN, TRO, UMOD
miR-582-5p	ACE2, ADRA1A, AHCYL2, AK021933, AKAP9, CAPN3, CXorf1, CYP2C19, DZIP3, EXOG, FEZF2, GBF1, GIT1, GNA14, GPRASP1, HIST1H1B, HLA-DRB6, Hs.587799, HTR7P1, ITIH5, KRT86, LOC729806, MAGT1, MLL, MRPS18C, NFATC1, NFKBIA, NPY2R, OGT, PCIF1, PIK3R4, PJA2, PRDM16, PSPH, SAG, SDC2, SH3TC1, SNAPC3, SRC, SUPV3L1, TBCE, TCP1, TRIM31, TYRO3, WSB1, WWOX, YTHDC2, ZNF257, ZNF639
miR-886-3p	ABAT, ANXA9, BTN2A1, BTN3A1, C14orf113, CARS, CDC42EP3, EFS, EHBP1, EPHB2, FEZ2, FGFR3, FKBP4, GRK5, ITGB7, LDHA, LPHN1, LY96, MAPT, MET, MICAL2, MSN, NAT15, NRP1, NT5E, PARD6A, PARN, PDE4D, PDLIM5, PGM1, PLAUR, POPDC3, PRKCA, PRLR, RABGAP1L, RHOD, RRBP1, RTCD1, SEC16A, SMPD1, SPATA2L, SRI, TDG, TGFBI, TJP3, UPP1, ZBTB3
miR-886-5p	AATK, ABCA2, ADAMTSL3, AMFR, BAT2L, BCL10, BRF2, BTN2A1, C14orf113, C8orf51, CD55, CD58, EHBP1, ENSG00000212670, EPHB2, EXPH5, F7, FEZ2, GTF3C1, HOXC10, Hs.598827, Hs.662238, ITGB7, LMF1, LPHN1, LY96, MAPT, MAZ, MICAL2, MXD4, NRP1, PARN, PAX3, PDCD6, PDE4D, PDGFB, PGM1, POPDC3, RABGAP1L, RTCD1, ST3GAL6, STK10, TBXA2R, TEAD1, TNFRSF4, XPNPEP3, ZNF638
miR-934	ADRA1A, AHSG, AK021724, AK021988, ALAS2, ALDH18A1, ALDH1A3, ANKMY1, AR, ARG2, ATP6V0A1, AV647366, CACNG3, CCKAR, CSGALNACT1, CTSS, CXCR3, ETS2, HNRNPCL1, HPR, Hs.663098, IBSP, ICA1, IFNA16, ITPR2, KIAA1731, KNTC1, LDB1, LGALS3BP, LILRB4, MAN1A2, NM_015858, PIPOX, POMZP3, POMZP3, PRMT7, SGEF, SLC25A12, SOD2, SOX2, SQSTM1, SV2A, TP53AIP1, TRADD, TSPAN2, TXNRD1, ZNF432, ZNF500, ZNF669
miR-1290	ADRA1A, AFAP1, AKAP10, ANXA4, AV647366, AVPR1A, C11orf10, CAMLG, CSGALNACT1, CYP4F3, FAM63A, FBLN1, HNRNPCL1, HTRA2, KCNG1, KDM3B, KLF5, KRT86, LGALS3BP, MAG, MKLN1, NFKB2, NPY2R, PIGB, RALB, RBP4, RELB, RP1-21O18.1, RPS6KA2, SFRS12, SNX2, SQSTM1, STX7, SV2A, TAX1BP1, TCP1, TCTN3, TEX261, TGOLN2, TPRA1, TRIM68, TXNRD1, UBQLN4, UBR5, UGP2, UQCRB, ZFAND5

Table S3. MicroRNAs (miRNAs) with overlapping targets between the identified messenger RNAs (mRNAs) and the predicted mRNAs.

miRNA	Targets
miR-29a	PDGFB,SPTLC2
miR-31	SOX11, STAU2, TMEM43
miR-125b	MAPRE2, PMM2
miR-1290	SFRS12
miR-130a	SLC6A6, TRIM3
miR-138	SH2B3
miR-140-3p	JAG1, SLC5A3
miR-141	PALM2-AKAP2
miR-143	FLJ10357, RALGPS1
miR-145	NUAK1
miR-155	SHANK2
miR-199a-3p/miR-199b-3p	FUT9, PATZ1
miR-199a-5p	FUT9, PIK3CD, SERPINE1
miR-200a	TNPO1
miR-200b	HEG1, RANBP10
miR-203	C5orf30, LPHN1, LPIN1, NFIB, PDE4D, ZFHX4
miR-205	MED13L
miR-376c	CDH11
miR-455-3p	GTPBP1
miR-582-5p	PRDM16
